# Metabolic reprogramming and prognostic modeling in pancreatic cancer: insights from WGCNA

**DOI:** 10.3389/fgene.2025.1487046

**Published:** 2025-06-12

**Authors:** Zhuo Song, Zhijia Sun, Yupeng Di, Xu Liu, Xiaoli Kang, Gang Ren, Yingjie Wang

**Affiliations:** ^1^ Department of Radiotherapy, Air Force Medical Center, Air Force Medical University, Beijing, China; ^2^ Department of Radiotherapy, Peking University Shougang Hospital, Beijing, China

**Keywords:** pancreatic cancer, predictable model, metabolism, DNA damage repair, bioinformatics

## Abstract

**Purpose:**

Metabolic reprogramming plays a crucial role in multiple malignant features of pancreatic cancer (PC). However, few studies have comprehensively examined metabolic features of PC and provided guidance for their treatment.

**Methods:**

This study tried to identify metabolism-associated hub genes based on metabolic phenotypic levels through weighted gene co-expression network analysis, and constructed a risk model for PC, then verified its accuracy and explored the potential mechanisms.

**Results:**

We screened out five metabolic hub and prognostic genes (*DLX3, HMGA2, SPRR1B, MYEOV*, and *FAM111B*) and constructed a novel metabolism-associated gene signature to predict the prognosis of PC. The model was verified efficacy and demonstrated with good performance through analysis of Kaplan-Meier plotter, receiver operating characteristic curves, comparing with reported models, application in predicting drug sensitivity and constructing a nomogram model. Correlation analysis revealed a close association between the levels of risk score and DNA damage response (DDR, correlation coefficient: 0.41, *P* < 0.001). Enrichment analysis indicated that risk scores were derived from multiple metabolic or proliferative pathways, providing further evidence that metabolism may mediate DDR to affect PC survival.

**Conclusion:**

Through bioinformatics analysis, we identified five prognostic relevant differentially expressed genes highlighting the role of metabolism-associated factors in pancreatic cancer, which reveals a strong correlation ship with DDR, offering new insights into treatment strategies that combine metabolism with DDR.

## 1 Introduction

Pancreatic cancer (PC) is one of the most lethal malignancies, accounting for 4.8% of all cancer-related deaths globally (Bray et al., 2024). Furthermore, the incidence and mortality rate of PC are increasing simultaneously. PC is predicted to become the second leading cause of cancer-related deaths worldwide by 2030 (Rahib et al., 2014). The standard treatment regimens for PC mainly include surgery, chemotherapy, radiotherapy. Despite recent advancements in these approaches, the prognosis for PC remains poor. This poor prognosis can be attributed to its aggressive nature, which leads to late-stage diagnosis, early metastases, and therapy resistance (Singhi et al., 2019). Therefore, gaining a comprehensive understanding of the aggressive features is crucial for investigating effective therapies in PC.

The invasiveness of PC is primarily determined by biological features such as extensive dense desmoplasia, hypoperfusion, and immune suppression (Hruban et al., 2019). Accumulating evidence suggests that metabolic alterations play a vital role in these malignant features and have emerged as a key hallmark of cancer. Malignant cells can alter their material and energy metabolism patterns to meet the requirements for survival and rapid proliferation (Hönigova et al., 2022); this process is known as metabolic reprogramming. The main metabolic pathways currently being studied include “Warburg effect”, “Glutamine addiction” and lipid metabolism (Chang et al., 2022; Xu et al., 2020; Man et al., 2020). The tricarboxylic acid (TCA) cycle and nucleic acid metabolism are intermediate and ultimate processes that link different metabolic patterns. Metabolic pathways determine cellular plasticity of changing and adapting to different microenvironments (Uddin et al., 2024). DNA damage repair (DDR) serves as maintaining genome integrity and is closely associated with metabolism (Li et al., 2022). Targeting nucleotide or amino acid metabolism can enhance the sensitivities of anti-tumor therapy by restricting DDR response (Falcone et al., 2022; Helleday and Rudd, 2022; Fu et al., 2019). A range of metabolic inhibitors has been developed and demonstrated promising anti-tumor potential in preclinical studies. For instance, the glycolysis inhibitor Lonidamine and the amino acid transporter inhibitor JHP 203 can synergistically enhance the therapeutic effect when combined with chemotherapy and radiotherapy, respectively (Tufail et al., 2024). However, challenges such as metabolic plasticity and the need for combination therapies persist, highlighting the urgency for continued research in this dynamic field.

However, the metabolism of PC is complex and interacts. Bioinformatics research provides an efficient means of interpreting clinical outcomes from metabolic mechanisms. A study attempted to demonstrate the complex plasticity mechanism of PC through a comprehensive analysis of genomics, transcriptomics, and proteomics, demonstrating the complex plasticity mechanism of PC, provides a case for us (Cancer Genome Atlas Research NetworkCancer Ge nome Atlas Research Network, 2017). Recently, several studies have reported the construction of metabolism-related models for PC, including from hypoxia, immunity, neuroendocrine, lactate, or lipid metabolism, suggested a link between metabolism and survival (Ma et al., 2021; Ye et al., 2021; Huang et al., 2023; Zhang et al., 2023; Yang et al., 2024; Peng et al., 2024). However, the methods used to screen genes for constructing these models only considered molecular-level factors and did not incorporate individual metabolic functional phenotypes.

This study aimed to apply weighted gene coexpression network analysis (WGCNA) to identify metabolism-associated hub genes and construct a metabolic risk model for PC and explore its potential mechanisms. Our study findings will enhance our understanding of the metabolic features of PC and facilitate the exploration of new therapeutic strategies combining with metabolism.

## 2 Materials and methods

### 2.1 Data collection and processing

**FIGURE 1:**
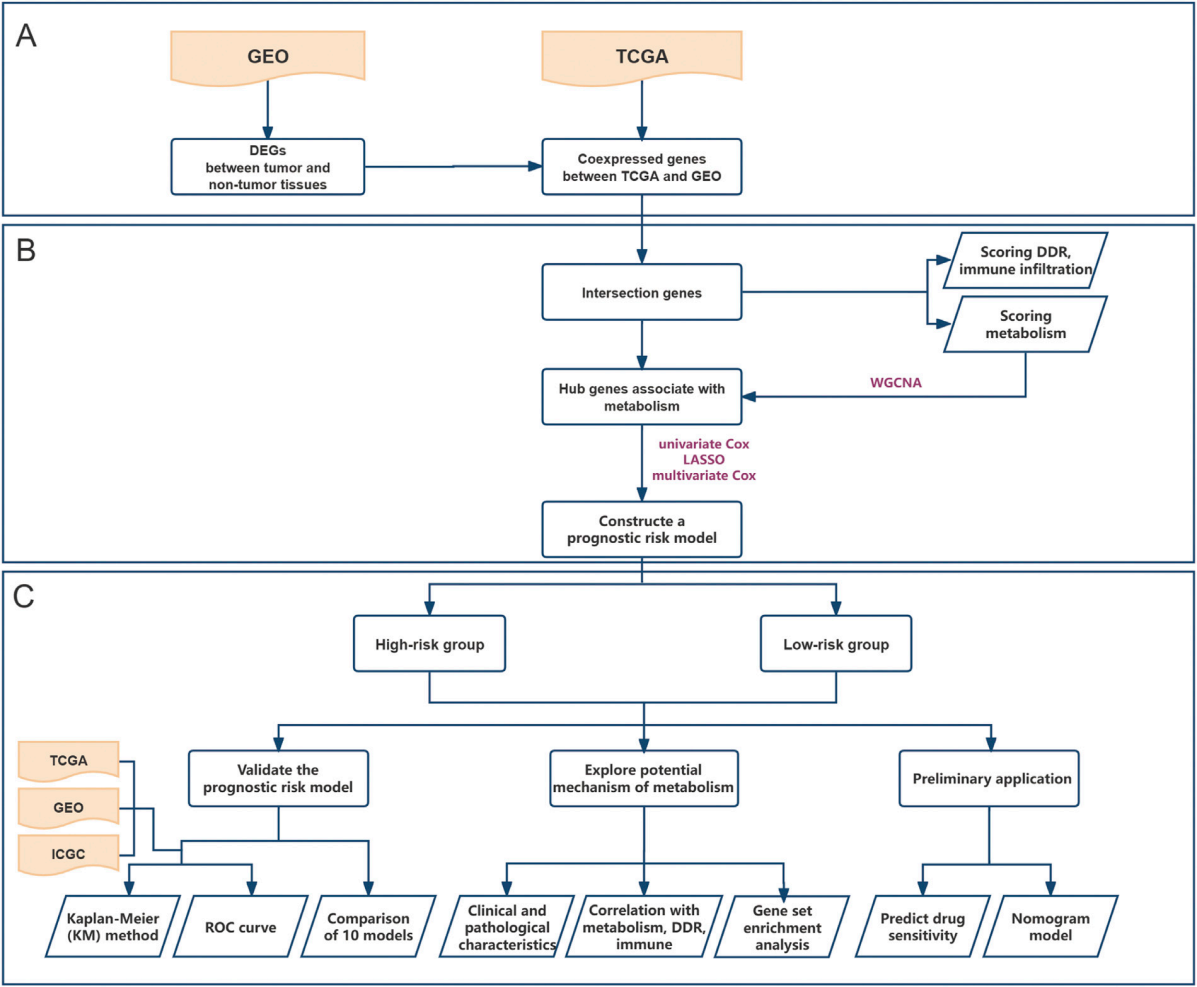
Flowchart depicting the whole study. **(A)** Screen for differentially expressed genes in pancreatic cancer based on GEO cohort and select a gene set that is co-expressed with TCGA; **(B)** Identify a metabolism-associated hub genes through WGCNA and construct a prognostic risk model; **(C)** Validate the model’s performance, explore underlying mechanisms, and further evaluate its application based on drug sensitivity analysis and clinical nomogram model.

The training and investigation dataset was obtained from the Cancer Genome Atlas (TCGA) database (https://portal.gdc.cancer.gov). Two additional datasets were obtained from the Gene Expression Omnibus (GEO; GSE62452) (https://www.ncbi.nlm.nih.gov/gds/) and International Cancer Genome Consortium (ICGC; https://icgc.org/) databases that served as external validation sets (Figure 1). All three datasets are publicly available and accessible online. The reasons for selecting these datasets for study were that they originated from PC patients, encompassed survival and transcriptional information, and boasted a sufficient sample size. After downloading the data, the following three steps were performed during the initial data processing: Patients with missing or zero overall survival (OS) were excluded, the transcriptome data from TCGA and ICGC databases were unified as log_2_ (x+1), and gene IDs were converted to gene symbols.

### 2.2 Scoring metabolism, DDR, and immune infiltration features

To assess the activity of different phenotypes, co-expressed genes were initially selected both in TCGA and GEO. To eliminate non-significant genes, DEGs between tumor and non-tumor tissues were then screened using the “limma” package in GEO, with a false discovery rate (FDR) < 0.05 as the cutoff. The intersection gene dataset, both in co-expressed genes and DEGs, was taken for subsequent analysis. The activities of different metabolic pathways and DDR for each sample were scored using the single-sample gene set enrichment of the R package GSVA in TCGA, utilizing the expression data of the intersection gene dataset. The metabolic signature gene set, which includes seven metabolic pathways (lipid, carbohydrate, amino acid, integrated energy, nucleotide, vitamin cofactor, and TCA), was previously reported (Sung and Cheong, 2021). In total, 276 DDR gene sets were obtained from previous studies and used for the knowledge-based curation of DDR pathways (Li et al., 2022). Immune infiltration analysis included the immune cell profiling (ICI) and estimate algorithm with the intersection gene dataset. ICI involved 22 types of immune cells using the “CIBERSORT” package, while the estimate algorithm included four types of scoring for immune, stromal, estimate, and tumor purity using the “ESTIMATE” package (Liu et al., 2022).

### 2.3 Identification of hub genes associated with metabolic phenotypic features using WGCNA

WGCNA was used to identify potential hub genes that share similar functions by constructing a co-expression network analysis and mining modules (Su et al., 2021). We applied WGCNA to define gene modules associated with metabolic phenotypic features derived from the intersection gene dataset in TCGA. Specifically, samples with outliers were removed first, and the function power estimate was used to define the optimal soft threshold. The subsequent steps and parameters of WGCNA included constructing correlation networks, setting the maximun number of gene modules to 30, and calculating the dissimilarity of the module eigengenes. The similarity cutoff was set to 0.75, and the modules with the top three high correlation coefficients were considered most relevant to metabolic features. These modules were merged for downstream analysis (Xiao et al., 2022).

### 2.4 Construction and performance analysis of prognostic risk model

For the training set, we used patient data from TCGA, whereas the validation sets consisted of data from GEO and ICGC. Univariate Cox regression using the “survival” package was employed to preliminarily filter the genes linked to the prognosis from the aforementioned metabolism-associated hub genes, with a p-value <0.001. Subsequently, LASSO regression with the “glmnet” package was used to address overfitting among the survival-related hub genes. Multivariate Cox stepwise regression was applied using the “survival” package to construct the prognostic model with the highest discriminatory ability for predicting OS. The equation for the risk score, which predicts prognosis based on metabolic hub genes, is shown in Figure 3A. The acquired formula for calculating the risk score was as follows: Risk score = (expression of *DLX3* × 0.114) + (expression of *HMGA2* × 0.144) + (expression of *SPRR1B* × 0.071) + (expression of *MYEOV* × 0.104) + (expression of *FAM111B* × 0.173).

Each patient was assigned a risk score and then categorized into high- and low-risk groups based on the median value independently within each cohort. To validate the accuracy of the model, survival curves were generated using the Kaplan–Meier (KM) method for the two groups separately in three cohorts. Additionally, the receiver operating characteristic (ROC) curve and area under the curve (AUC) values were calculated using the “survival ROC” package to evaluate the time-dependent predictive ability of the model. Furthermore, we compared our model with nine reported models from the literature in terms of their ability to predict 1-year survival, using ROC and AUC analysis (Ma et al., 2021; Ye et al., 2021; Huang et al., 2023; Zhang et al., 2023; Ren et al., 2021; Liu et al., 2021; Yan et al., 2020; Yan et al., 2022; Zhou et al., 2019).

### 2.5 Characteristics between the two risk groups

We compared the differences between the two groups in terms of available characteristics, including age, stage, tumor site, therapies, alcohol history, and the expression of the five indicative genes in TCGA. The comparison results were presented using boxplots or heatmaps generated by the “ggpubr” and “Complex Heatmap” packages.

### 2.6 Correlation analysis between risk score and phenotypic features

To explore the underlying mechanism of the risk score, we analyzed the association between risk scores and phenotypic features, including metabolism, DDR, and immune infiltration. This analysis involved comparing the differences in seven metabolic pathways, 22 immune cell infiltrations, and four types of estimates between the two different risk groups. Additionally, we analyzed the correlation between significantly different phenotypic features. The results were visualized using violin charts, beeswarm plots, or correlation heatmaps generated by the “ggplot2,” “ggbeeswarm,” and “corrplot” packages.

### 2.7 Gene set enrichment analysis

To further investigate the implied mechanisms of the risk score, gene set enrichment analysis (GSEA) was performed on DEGs between two risk groups. The DEGs were initially screened using the “DESeq2” package, with a |log2FC| > 1 and an adjusted FDR <0.05 as the cutoffs. The direction of regulation (up or down) for the DEGs was plotted using the “ggplot2” package. DEGs with an adjusted FDR <0.05 were then used for GSEA. Gene Ontology (GO) enrichment analysis, Kyoto Encyclopedia of Genes and Genomes (KEGG) pathway analysis, and GSEA were performed using the “Clusterprofiler” package with reference to “org.Hs.eg.db” (Carlson M, 2019. org.Hs.eg.db: Genome wide annotation for Human. R package version 3.8.2.). An adjusted p-value <0.05 was selected as the cutoff criteria for considering significant results in GO terms, KEGG pathway, and GSEA.

### 2.8 Drug sensitivity analysis and validation

The drug sensitivity of patients was predicted using the half-maximal inhibitory (IC50) calculated with the “pRRophetic” package. We selected several recommended or associated drugs for drug sensitivity analysis based on the clinical guidelines of PC. The difference between the two groups was assessed and displayed using columnar beeswarm plots created with the “ggbeeswarm” and “customLayout” packages. Additionally, we downloaded data on patient response to gemcitabine in TCGA using the “TCGAbiolinks” package, as reported by Nicolle, and validated it between the two groups (Nicolle et al., 2021).

### 2.9 Construction of a clinical nomogram

To demonstrate the practical application, a clinical nomogram model combining the risk score and clinical parameters was constructed using the “survival” and “RMS” packages. Owing to the large number of missing values for clinical parameters in TCGA, only variables (age, stage, radiotherapy, and pharmatherapy) with a sufficient number of samples and high relevance to survival were included. The nomogram was used to evaluate the 1-, 2-, and 3-year survival rates of patients in TCGA. Calibration curves and the C-index were used to assess the consistency between the predicted and actual survival rates.

### 2.10 Statistical analysis

Statistical analysis was performed using GraphPad Prism (version 9.0.0 for Windows) and R software (version 4.2.1 for Windows). In GraphPad Prism, *t*-tests or Wilcoxon signed-rank tests were used to compare measured differences between groups, and the chi-square test was used to analyze associations between categorical variables. Other statistical analyses were primarily conducted using R software, as described earlier, including scoring each feature, identifying DEGs and hub genes, GSEA, calculating drug sensitivity, and constructing the nomogram. The correlation coefficient between phenotypic features was evaluated using Pearson’s correlation. Statistical significance was set at *P* < 0.05.

## 3 Results

### 3.1 Identification of metabolism-associated hub genes in PC

We obtained transcriptome and clinical data from 178 PC patients (via TCGA) in this study for constructing models and exploring mechanisms. Furthermore, we obtained an additional 159 PC cases (69 in GSE62452 and 90 in ICGC-PAAD-US) for validating the risk model. To screen DEGs in GSE62452, we collected 61 non-tumor tissues from PC patients. We identified 10,031 genes that existed in both TCGA and GSE62452, and 6,747 genes were screened as DEGs in GSE62452. For further analysis, we obtained 5,872 genes as the intersection gene dataset, and their transcriptome data were used to score metabolism, DDR, and ICI activities for each patient.

**FIGURE 2:**
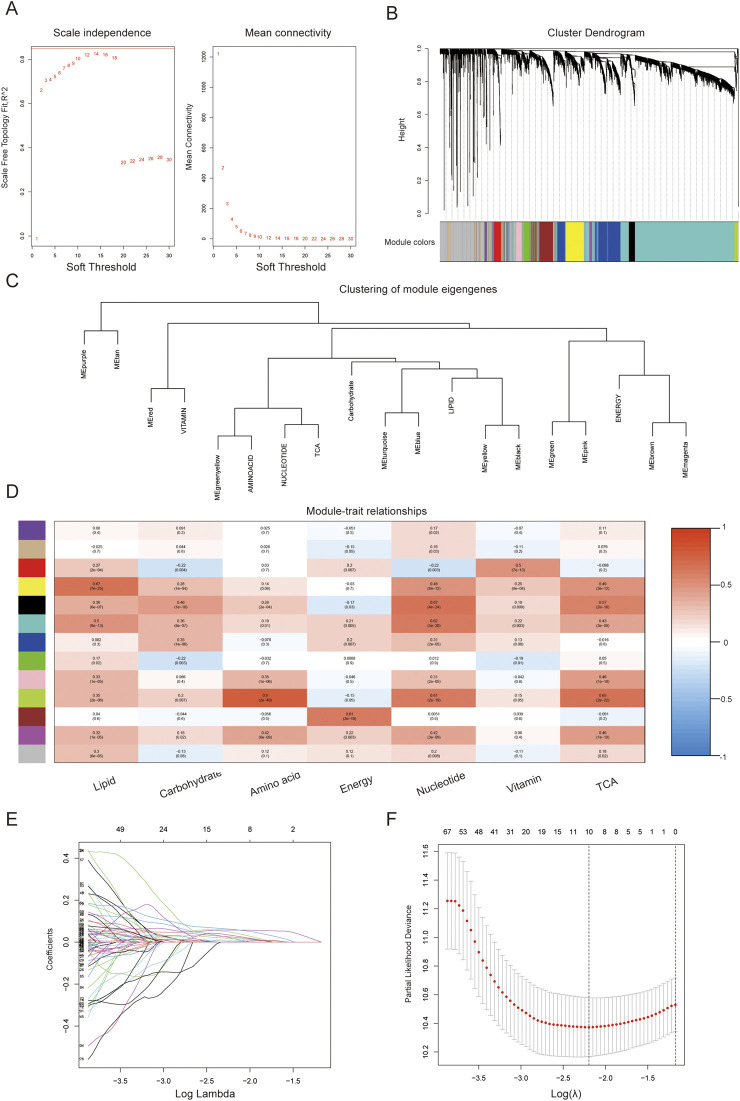
Identification of hub predictive genes associated with metabolic features using WGCNA. **(A)** Analysis of scale independence and mean connectivity for the soft thresholding powers; **(B)** Cluster dendrogram; **(C)** Clustering of module eigengenes; **(D)** Correlation of the gene module with metabolic phenotypic features (The data in the module represents the correlation coefficient and statistical value between each gene set and metabolic pathway); **(E)** Confidence intervals of log lambda; **(F)** Partial likelihood deviance of log Lambda.

The function power analysis showed that a soft threshold of 14 was optimal for ensuring a scale-free network in the WGCNA (Figure 2A). By merging similarity modules and applying a cut-line, we identified a total of 13 modules, with each module assigned a color using the dynamic tree cut method (Figure 2C). The coexpression network was visualized in a network heatmap plot (Figure 2B). Each color module represented a gene set with closely associated expressions. The gray module represented genes that were not assigned to any other cluster. The other 12 modules were plotted in a heatmap to evaluate the association between each module and different metabolic pathways (Figure 2D). Based on the criteria (|Cor| > 0.65), yellow, black, and green-yellow modules, which collectively included 536 genes, were selected for further analysis.

### 3.2 Construction and validation of a metabolism-associated prognostic risk model

To filter genes with predictive value, we performed univariate Cox regression analysis and identified 362 genes among the metabolic hub genes (p < 0.001). Based on the 1000-fold cross-validation results of LASSO regression analysis, the optimal efficacy was achieved when ten variables were selected as the target markers (Figures 2E,F). Furthermore, through multivariate Cox regression analysis, we identified five genes derived from the LASSO regression analysis that could independently construct a gene signature to predict the prognosis of PC as described in 2.4.

**FIGURE 3:**
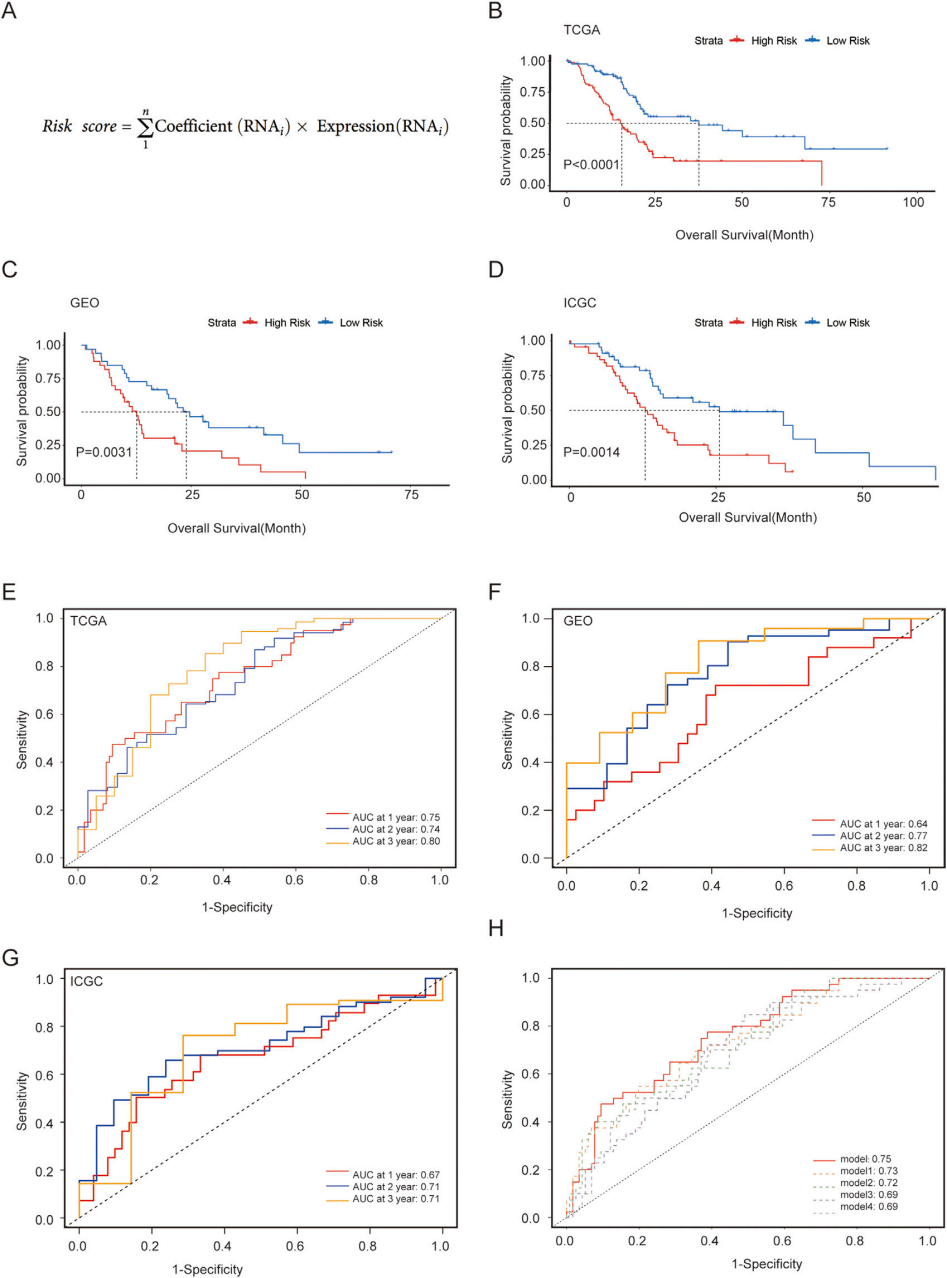
Construction and validation of a metabolism-associated prognostic risk model. **(A)** Risk score for the model; **(B–D)** Survival curves analysis of three cohorts; **(E–G)** ROC curve and evaluated AUC values for three cohorts; **(H)** Comparison of our model with four other models demonstrating superior predictive efficacy.

Patients were divided into two groups based on the median risk score for validation. The KM curve showed that the high-risk group had significantly worse OS than the low-risk group (Figure 3B), which was further verified in external validation sets from GEO and ICGC (Figures 3C,D). The ROC curves and AUC values of the three datasets demonstrated the predictive ability of the risk score from a time-dependent perspective (Figures 3E–G). In TCGA, the AUCs for 1-, 2-, and 3-year were 0.75, 0.74, and 0.80, respectively, indicating good predictive performance for PC. When compared with other reported models, our model exhibited a better ROC curve and AUC value, and the top five models are displayed in Figure 3H (Supplementary Figure S1).

### 3.3 Clinical and pathological characteristics of two groups

**FIGURE 4:**
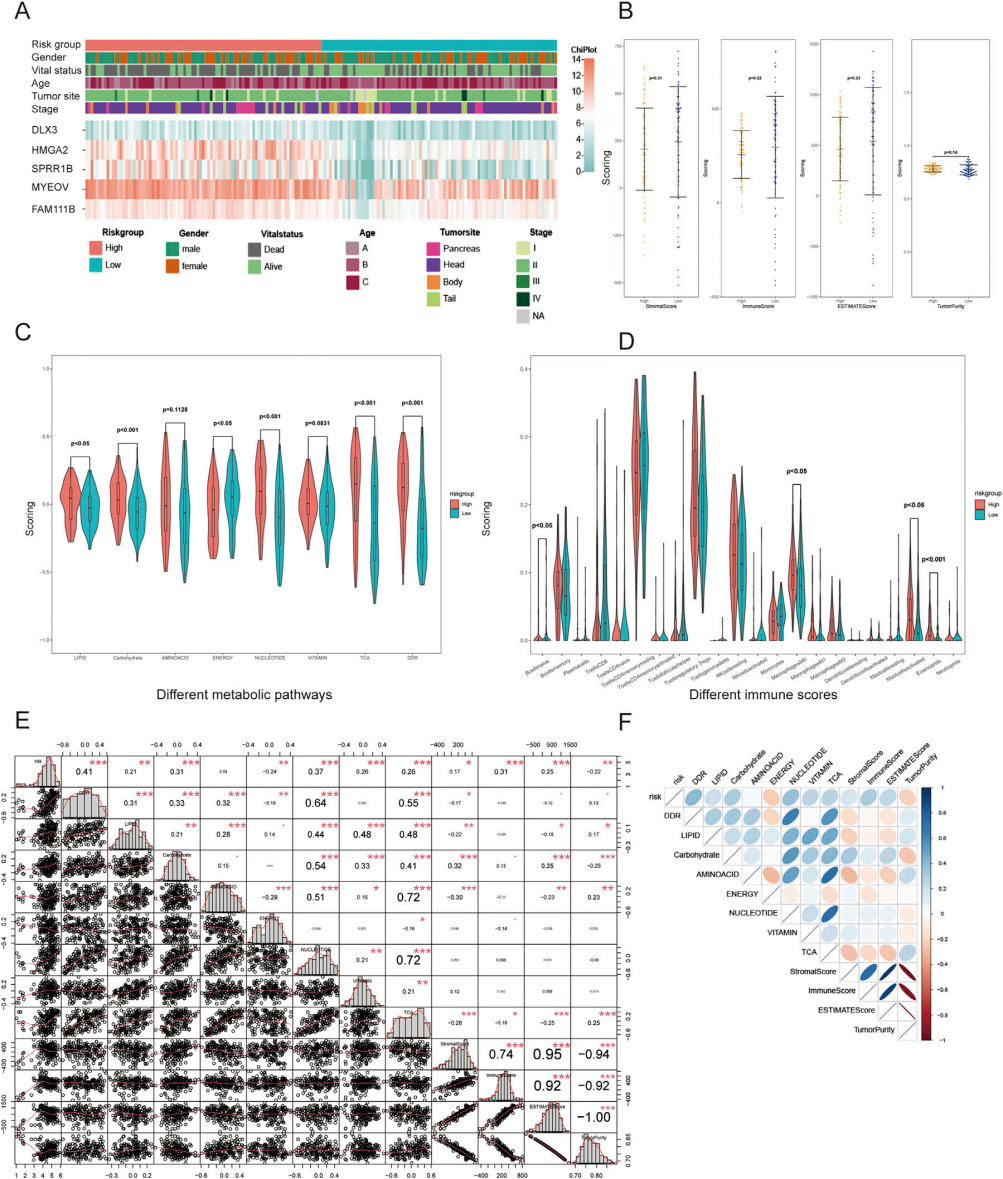
Differences in clinical and phenotypic features between the two groups. **(A)** Heatmap of clinical features and gene expression; **(B)** Differences in estimate scores; **(C)** Differences in seven metabolic pathways and DDR; **(D)** Differences in 22 immune cell infiltrations; **(E)** Correlation coefficients between significant phenotypic features; **(F)** Heatmap of the correlations. *p < 0.05, **p < 0.01, ***p < 0.001.

The distribution of clinical and pathological information between the two groups is presented in Table 1. The analysis results showed that, except for vital status and pathological types, no significant distribution differences were observed in other characteristics. The expression levels of the five indicator genes in the high-risk group were higher than those in the low-risk group, which may explain the potential mechanisms of OS (Figure 4A).

**TABLE 1:** Clinical information of the two groups in TCGA.

Characteristic	Clinical information in TCGA cohort			P value
	High-risk group (n = 89)	Low-risk group (n = 89)	Overall (n = 178)	
Age (years)				
Mean (SD)	63.49 (11.34)	65.61 (10.30)	64.55 (10.88)	0.1974
Median [Min, Max]	64 [41,85]	66 [35,88]	65 [35,88]	
Gender				
Male	48 (54%)	50 (56%)	98 (555)	0.7631
Female	41 (46%)	39 (44%)	80 (45%)	
Vital status				
Alived	29 (33%)	56 (63%)	85 (48%)	<0.0001
Dead	60 (67%)	33 (37%)	93 (52%)	
Pathological type				
Infiltrating duct carcinoma	78 (88%)	64 (72%)	142 (80%)	0.0314
Adenocarcinoma	8 (9%)	13 (15%)	21 (12%)	
Neuroendocrine carcinoma	3 (3%)	8 (9%)	11 (6%)	
Mixed or others	0 (0%)	4 (4%)	4 (2%)	
Stage				
I	7 (8%)	14 (16%)	21 (12%)	0.3757
II	77 (87%)	69 (78%)	146 (82%)	
III or IV	4 (4%)	4 (4%)	8 (4%)	
Unknown	1 (1%)	2 (2%)	3 (2%)	
Tumor site				
Head of PC	65 (73%)	64 (72%)	129 (72%)	0.5290
Body of PC	5 (6%)	10 (11%)	15 (8%)	
Tail of PC	8 (9%)	7 (8%)	15 (8%)	
Pancreas	11 (12%)	8 (9%)	19 (11%)	
Pharmaceutical Therapy				
Yes	67 (75%)	63 (71%)	130 (73%)	0.6456
No	17 (19%)	22 (25%)	39 (22%)	
Unknown	5 (6%)	4 (4%)	9 (5%)	
Radiation Therapy				
Yes	21 (24%)	27 (30%)	48 (27%)	0.5561
No	60 (67%)	56 (63%)	116 (65%)	
Unknown	8 (9%)	6 (7%)	14 (8%)	
Alcohol history				
Yes	55 (62%)	46 (52%)	101 (57%)	0.2235
No	27 (30%)	38 (43%)	65 (37%)	
Unknown	7 (8%)	5 (6%)	12 (7%)	

### 3.4 Correlation between risk score and phenotypic features

The risk score showed strong associations with metabolism, DDR, and estimate scores but weak correlations with 22 immune cells, as revealed by the differential analysis (Figures 4B–D). The high-risk group demonstrates lower scores of stromal, immune, and estimate, while the tumor purity score is higher. In terms of metabolism, the high-risk group exhibits higher scores for TCA, DDR and metabolism of lipid, carbohydrate, amino acid, nucleotide, vitamin, but a lower score for integrated energy metabolism. To further investigate the implied mechanism of the risk score, correlation analysis revealed that the risk score had the strongest correlation with DDR rating scores (correlation coefficient: 0.41, *P* < 0.001), followed by nucleotide, carbohydrate, immune, TCA, and vitamin scores (Figures 4E,F). In terms of the relationship between phenotypic features, DDR showed a strong correlation with lipid, carbohydrate, amino acid, nucleotide, and TCA metabolism, particularly with the latter two features. In contrast, the immune score displayed a weak correlation with metabolism and DDR. For the internal analysis of metabolism pathways, the correlation coefficient between nucleotide and TCA was 0.72, and both were closely related to lipid, carbohydrate, and amino acid metabolism. The correlation between different factors showed a positive correlation, except for energy metabolism. This reflects that patients identified as high-risk by our model may be more closely related to changes in the DDR pathway, which requires the support of metabolic pathways such as nucleotide and TCA.

### 3.5 GSEA reveals the mechanism of risk derived from multiple metabolic or proliferative pathways

**FIGURE 5:**
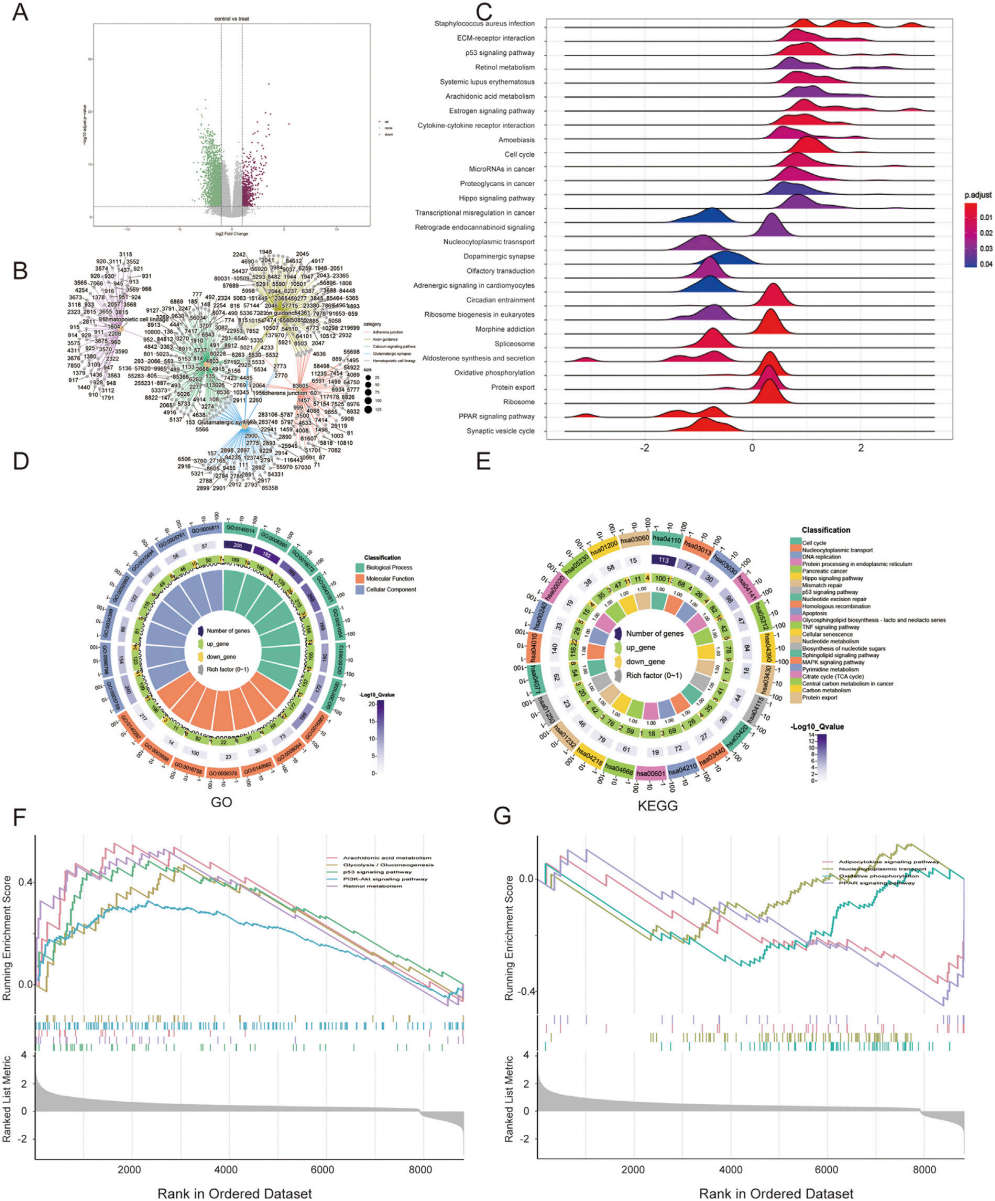
Gene set enrichment analysis (GSEA) between two risk groups. **(A)** Volcano plot of DEGs; **(B)** Connection between genes and pathways; **(C)** GSEA results; **(D)** GO analysis of DEGs; **(E)** KEGG analysis; **(F,G)** GSEA of upregulated and downregulated pathways.

In total, 10,720 DEGs were re-identified as potential factors that could contribute to the survival differences between the two different risk groups. Among them, 705 DEGs were upregulated and 2,660 DEGs were downregulated in the high-risk group compared with those in the low-risk group (Figure 5A). GO analysis of the DEGs indicated that these genes were highly enriched in biological processes such as nucleocytoplasmic transport or replication, cellular components including mitochondria, and molecular functions related to regulating metabolism or ATP-dependent activities, as shown in Table 2 (Figure 5D). KEGG analysis and GSEA results showed that DEGs were enriched in several pathways closely related to metabolism and proliferation (Figures 5B,C,E). Nine pathways may explain the factors associated with the risk score and their effect on survival; among these pathways, five were upregulated and four were downregulated (Figures 5F,G).

**TABLE 2:** GO analysis results of the DEGs.

Ontology	ID	Description	GeneRatio	p.Adjust	Count
Biological Process	GO:0140014	mitotic nuclear division	208/6734	6.47E-22	208
	GO:0006260	DNA replication	182/6734	3.79E-19	182
	GO:0016072	rRNA metabolic process	165/6734	1.72E-15	165
	GO:0043161	proteasome-mediated ubiquitin-dependent protein catabolic process	248/6734	5.46E-15	248
	GO:0051054	positive regulation of DNA metabolic process	169/6734	4.43E-11	169
	GO:0006913	nucleocytoplasmic transport	180/6734	1.54E-10	180
	GO:2001020	regulation of response to DNA damage stimulus	172/6734	1.96E-10	172
Cellular Component	GO:0005759	mitochondrial matrix	240/7017	1.74E-09	240
	GO:0098798	mitochondrial protein-containing complex	154/7017	5.41E-08	154
	GO:0034399	nuclear periphery	86/7017	9.16E-08	86
	GO:0032993	protein-DNA complex	122/7017	1.29E-07	122
	GO:0010494	cytoplasmic stress granule	55/7017	4.07E-07	55
	GO:0005761	mitochondrial ribosome	56/7017	1.98E-06	56
	GO:0005811	lipid droplet	57/7017	0.00016	57
Molecular Function	GO:0016887	ATP hydrolysis activity	199/6853	7.17E-12	199
	GO:0008094	ATP-dependent activity, acting on DNA	73/6853	1.54E-05	73
	GO:0140662	ATP-dependent protein folding chaperone	30/6853	0.000166	30
	GO:0008378	galactosyltransferase activity	23/6853	0.001299	23
	GO:0016758	hexosyltransferase activity	100/6853	0.001474	100
	GO:0003688	DNA replication origin binding	14/6853	0.001685	14
	GO:0140297	DNA-binding transcription factor binding	217/6853	0.00237	217

### 3.6 Drug sensitivity analysis and validation

**FIGURE 6:**
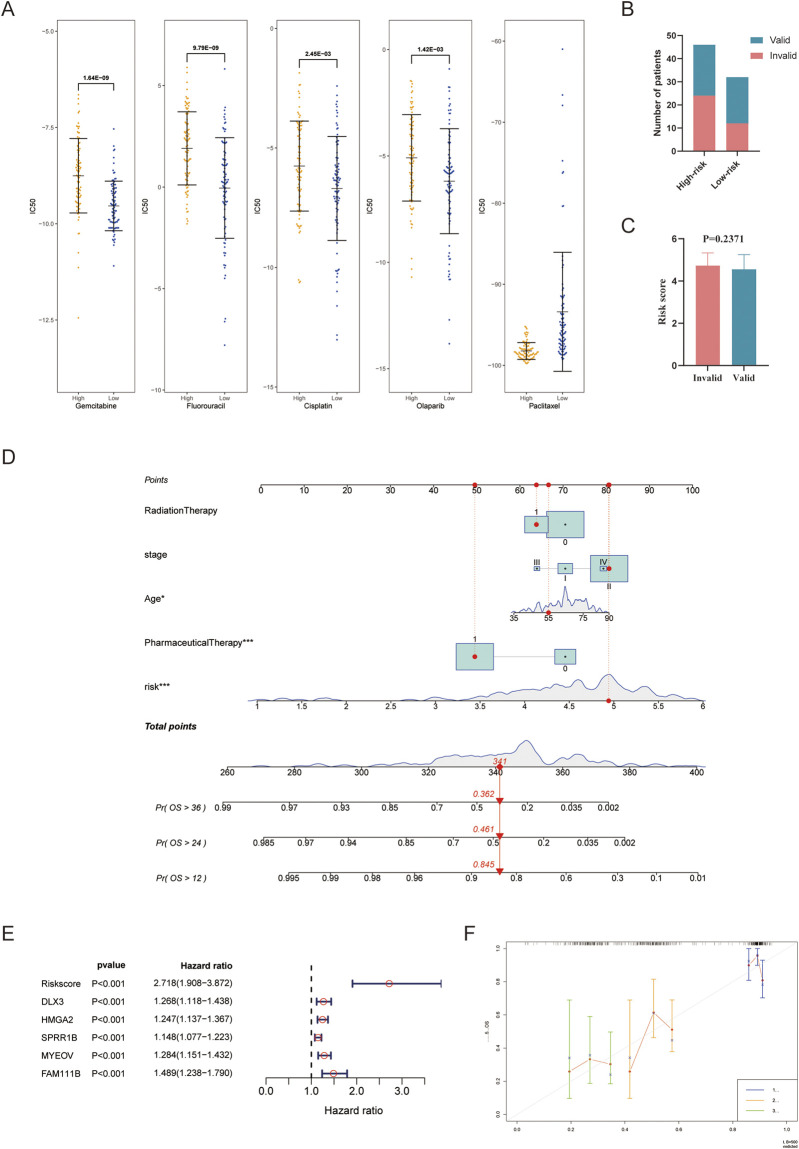
Predicting drug sensitivity and constructing a nomogram model. **(A)** Comparison of IC50 values between the high- and low-risk group; **(B,C)** Analysis of actual response to gemcitabine; **(D)** Nomogram model integrating the risk score and valuable clinical parameters; **(E)** Univariate Cox analysis of the risk score and included genes; **(F)** Calibration curves for 1, 2, and 3 years of the nomogram. ***P < 0.001.

IC50 values reflect the sensitivity of patients to drugs, with lower values of IC50 indicating greater sensitivity. Gemcitabine and 5-fluorouracil were the only pharmaceuticals included in the drug sensitivity analysis that were also recommended in the PC guidelines. Additionally, the IC50 values of cisplatin, olaparib, and paclitaxel were analyzed considering that oxaliplatin and albumin paclitaxel were recommended in the PC guidelines. Furthermore, the predictive IC50 values of these five pharmaceuticals to assess the predictive value of the risk score between the two groups. The results showed that, except for paclitaxel, the IC50 values of other pharmaceuticals in the high-risk group were significantly higher than those in the low-risk group (Figure 6A). In the analysis of the actual response to gemcitabine, a trend emerged suggesting that patients with lower risk scores and a higher proportion in the low-risk group exhibited better responses (Figures 6B,C).

### 3.7 Application of risk score in a clinical nomogram model

To further validate the accuracy and usability of the risk score, a nomogram was constructed using the risk score and several clinical parameters. The total points obtained from the nomogram were used to evaluate the 1-, 2-, and 3-year OS rates in PC (Figure 6D). The nomogram displayed that younger age, administration of pharmaceutical therapy, and low-risk scores were all protective prognostic factors; these tendencies were consistent with expectations. The previous administration of radiotherapy and the later stage of the disease also showed a partial protective tendency. However, the sample size in some cohorts was insufficient to achieve statistical significance. A forest plot was used to display the predictive value of the five hub genes with hazard ratios in PC (Figure 6E). We further analyzed levels of five genes in other cancers with TCGAplot, most of them upregulated (Supplementary Figures S2A–E) (Liao and Wang, 2023). The protein expression of them on the Human Protein Atlas database may also upregulated in PC (DLX3 and SPRR1B pending analysis) (Supplementary Figures S2F,H). To verify the accuracy of the model, calibration curves were performed (Figure 6F), and the C-index was found to be 0.778 (0.729–0.828), indicating that the nomogram has good capability for predicting the OS rate of PC patients.

## 4 Discussion

Metabolic reprogramming plays a crucial role in the mechanisms for underlying many malignant behaviors of PC (Hao et al., 2021). Understanding its characteristics and exploring potential targets are of great significance for improving treatment efficacy. Numerous studies have demonstrated that targeting specific metabolic pathways can exert anticancer effects (Zu et al., 2022). However, the regulation process of metabolic reprogramming is complex, and few studies have comprehensively considered multiple metabolic pathways simultaneously. Therefore, we aimed to construct and validate a metabolism-associated prognostic risk model based on the level of metabolic phenotype, and explore its potential mechanisms.

Constructing a prognostic model is a common approach in bioinformatics analysis based on transcriptome data. In previous studies, directly intersected with phenotype-associated gene sets was a conventional step in constructing phenotype-related prognostic models. Metabolism has been a focus in similar studies. However, the gene sets used to construct models based on specific phenotypes that were often derived from previous studies. Our study adopted a method that screens hub genes based on metabolic phenotype scorings through using WGCNA. This approach recognizes that functional phenotypes of patients are often the result of combined actions of sets of genes. The performance of our model was well validated using KM and ROC curves in external datasets and exhibited good feasibility in a nomogram model. Moreover, our model demonstrated superior predictive ability for 1-year survival compared with that of the nine reported PC models. Our study findings revealed a closer relationship between risk score, metabolism, and DDR levels than between immunological or clinical features. This can be reasonably explained by the role of metabolism in mediating multiple phenotypes of PC that affect survival, particularly through DDR. As summarized in previous reviews, metabolites of lipid, carbon, and amino acids serve as the primary sources for nucleic acid metabolism and interact through the TCA pathway, collectively establishing the basis for DDR (Hönigova et al., 2022; Jurkovicova et al., 2022). The analysis results of GO, KEGG, and GSEA indicated that the risk score may originate from multiple metabolic or proliferative pathways, reaffirming the significance of metabolic processes in various malignancies of PC.

Five genes (*DLX3, HMGA2, SPRR1B, MYEOV*, and *FAM111B*) were included in this model. In the survival analysis of these five genes, each of them was significant as a risk factor in univariate Cox regression, whereas only *HMGA2* was significant in multivariate Cox regression. Multivariate Cox analysis demonstrated that a robust prognostic risk model could be constructed using these integrated five genes. Except for *DLX3*, cumulative evidence has indicated that these genes participate in the malignant process of PC and are accepted for constructing similar models. As reported previously, *HMGA2* sever as an upstream protein that alters chromatin structure and regulates gene expression, participating in various tumorigenic process (Huang et al., 2018; Hashemi et al., 2023). A necroptosis-related signature was constructed with HMGA2, indicating the predictive value of HMGA2 in PC (Chen et al., 2022). FAM111B and MYEOV have been incorporated into a necroptosis‐related prognostic model for PC (Wu et al., 2022). Preliminary single-gene bioinformatics studies have indicated that FAM111B and MYEOV are highly expressed and closely associated with poor prognosis in PC (Gong et al., 2022; Tang et al., 2020). The enrichment results of FAM111B were related to cell cycle signaling and DDR pathways, while MYEOV was related to several glycolysis-related pathways. Emerging evidence indicates that overexpression of MYEOV increases the expression of metabolism-related enzyme genes through the c-Myc and mTORC1 pathways (Tange et al., 2023). SPRR1B has been validated in lung adenocarcinoma and included in a PC model (Zhang et al., 2021; Liu et al., 2019). A pan-cancer analysis revealed that SPRR1B was a significant biomarker of cancer stemness that used for predicting immunotherapy response (Zhang et al., 2022). The homeobox transcription regulator DLX3 is known for regulating skin epidermal homeostasis, and its loss induces squamous cell carcinoma of the skin (Bhattacharya et al., 2018; Palazzo et al., 2016). The mutation of DLX3 on autosome leads to tricho-dento-osseous syndrome (Dong et al., 2023). In our study, we found that DLX3 was a risk factor for PC, not a protective factor as observed in squamous cell carcinoma of the skin. Further studies are needed to determine whether this paradox can be attributed to the pathological characteristics of adenocarcinoma with abundant stroma in PC.

To our knowledge, this is the first study to screen metabolism-associated hub genes based on metabolic phenotype scoring and construct a prognostic model. Nonetheless, our study has some limitations. First, our data were from obtained online databases TCGA, GEO, and ICGA, but analysis data used for exploring phenotypic features, drug sensitivity, and nomogram model was only from TCGA. Second, real prospective clinical cohorts and basic investigations are needed for further validation. Lastly, the specific molecular mechanisms explaining the crosstalk between risk score, metabolism, and DDR are still poorly understood.

In conclusion, we constructed and validated a metabolism-associated prognostic model based on the levels of seven metabolic phenotypes in PC. The findings suggest that the mechanism of risk scores is primarily related to DDR and the metabolism of nucleotides, carbohydrates, and TCA. The close relation between metabolism and DDR may indicate that metabolic reprogramming provides abundant substances for DDR, promoting malignant behaviors. This offers new insight into the combined treatment strategies of PC.

## Data Availability

The original contributions presented in the study are included in the article/Supplementary Material, further inquiries can be directed to the corresponding authors.

## Supplementary material

SUPPLEMENTARY FIGURE S1 Comparison of ten models in TCGA dataset using ROC and AUC analysis. **(A)** Our model demonstrates superior predictive efficacy in PC; **(B)** Metabolism-related model reported by Huang et al.; **(C)** KRAS-associated metabolic model reported by Ma et al.; **(D)** Lipid metabolism-related model reported by Ye et al.; **(E)** Neuroendocrine regulation- and metabolism-related model reported by Zhang et al.; **(F)** Stem cell-related model reported by Ren et al.; **(G)** Immune-related model reported by Liu et al.; **(H)** Pyroptosis-related model reported by Yan et al.; **(I)** Four-gene model reported by Yan et al.; **(J)** Four-gene model reported by Zhou et al.

SUPPLEMENTARY FIGURE S2 The transcription levels of five genes in pan cancers **(A–E)** and protein expression of HMGA2 **(F)**, MYEOV **(G)**, and FAM111B **(H)** in cancer and paracancer tissue (A color version of this figure is available in the online journal.). **P* < 0.05, ***P* < 0.01, ****P* < 0.001, *****P* < 0.0001, ns, no statistically significant.

## Abbreviations

PC, pancreatic cancer; TCA, tricarboxylic acid cycle; DDR, DNA damage repair; DEG, differential expression genes; WGCNA, weighted gene co-expression network analysis; LASSO, least absolute shrinkage and selection operator; TCGA, the cancer genome atlas; GEO, gene expression omnibus; ICGC, international cancer genome consortium; OS, overall survival; IGDS, intersection genes data set; ICI, immune cell profiling; KM, Kaplan-Meier; ROC, receiver operating characteristic; AUC, area under the curve; GSEA, gene set enrichment analysis; FDR, false discovery rate; GO, gene ontology; KEGG, Kyoto encyclopedia of genes and genomes; IC50, half maximal inhibitory concentration.
