# Supplementary material for: Metabolic reprogramming and prognostic modeling in pancreatic cancer: insights from WGCNA
Source: Front Genet. 2025 Jun 12;16:1487046. doi: 10.3389/fgene.2025.1487046 (PMC12198206; doi:10.3389/fgene.2025.1487046)

Supplementary Figure 1. Comparison of ten models in TCGA dataset using ROC and AUC analysis. (A) Our model demonstrates superior predictive efficacy in PC; (B) Metabolism-related model reported by Huang et al.; (C) KRAS-associated metabolic model reported by Ma et al.; (D) Lipid metabolism-related model reported by Ye et al.; (E) Neuroendocrine regulation- and metabolism-related model reported by Zhang et al.; (F) Stem cell-related model reported by Ren et al.; (G) Immune-related model reported by Liu et al.; (H) Pyroptosis-related model reported by Yan et al.; (I) Four-gene model reported by Yan et al.; (J) Four-gene model reported by Zhou et al.


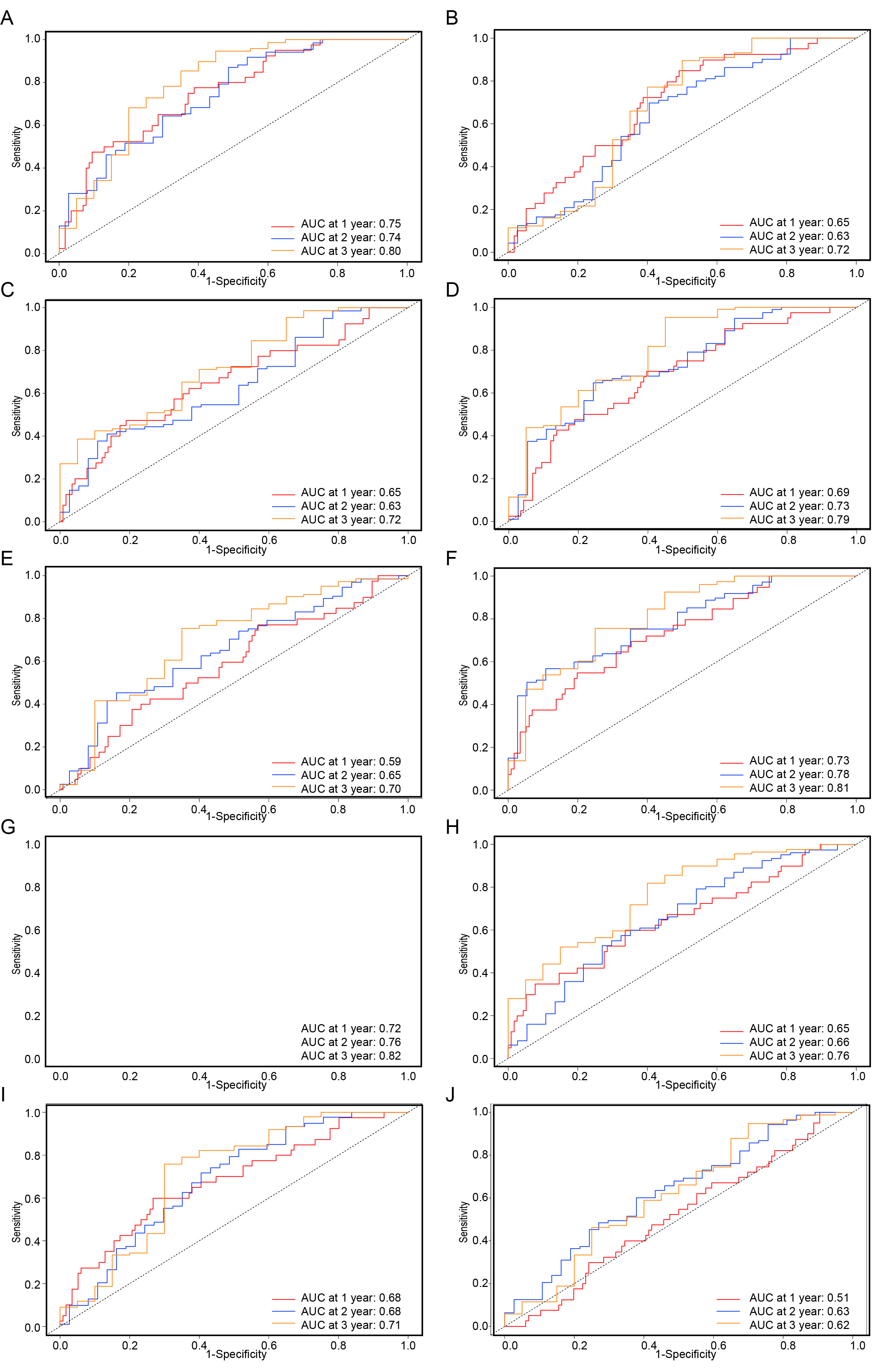


Supplementary Figure 2 The transcription levels of five genes in pan cancers (Sup fig 2A-E) and protein expression of HMGA2 (Sup fig 2F), MYEOV (Sup fig 2G), and FAM111B (Sup fig 2H) in cancer and paracancer tissue (A color version of this figure is available in the online journal.). **P* < 0.05, ***P* < 0.01, ****P* < 0.001, *****P* < 0.0001, ns, no statistically significant.


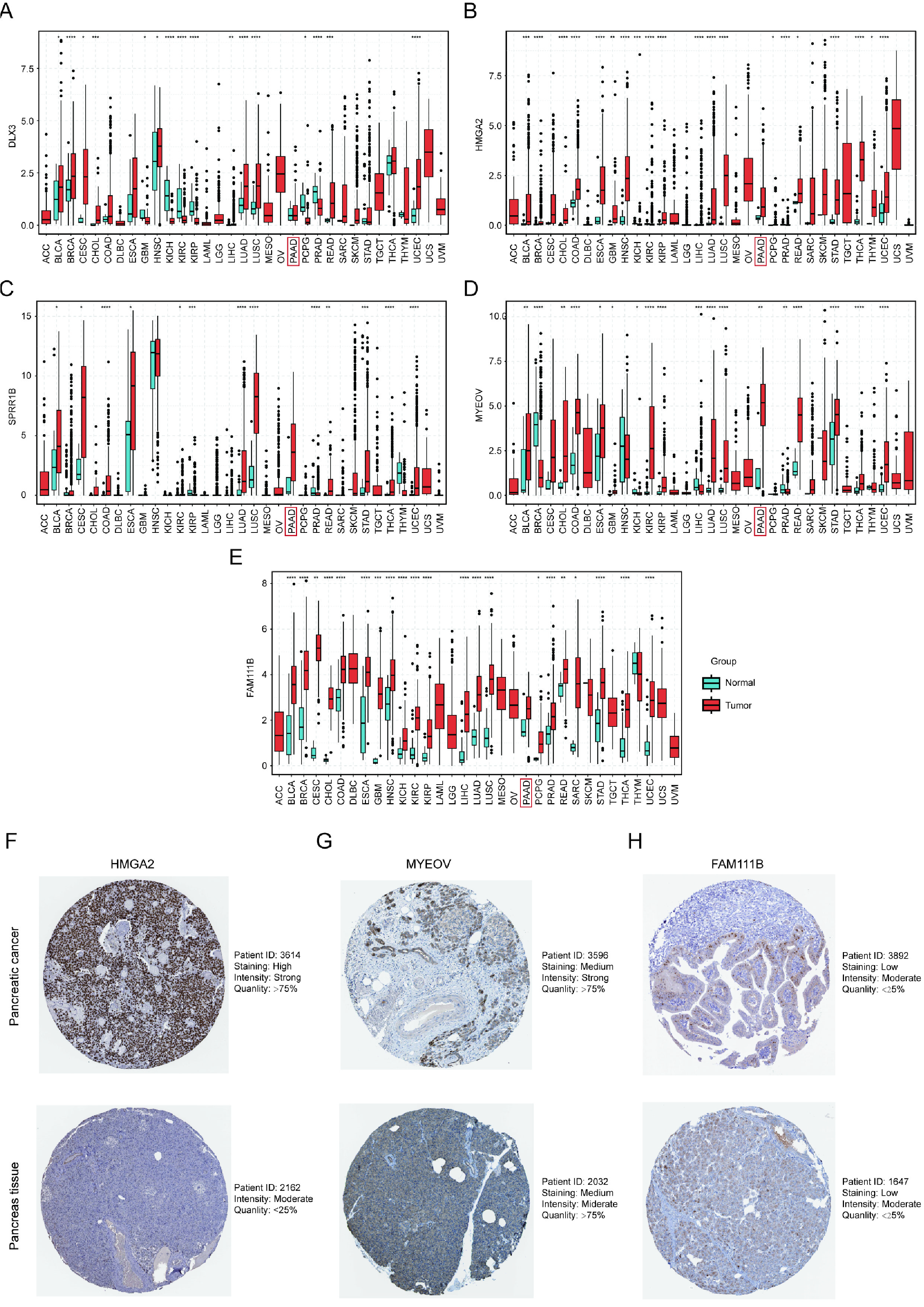

Supplement: Supplementary file 1 [file DataSheet1.docx]
